# Challenges and limitations in developing of a new maxillary standardized rat alveolar bone defect model to study bone regenerative approaches in oral and maxillofacial surgery

**DOI:** 10.3389/fbioe.2025.1494352

**Published:** 2025-08-04

**Authors:** Naïma Ahmed Omar, Jéssica Roque, Céline Bergeaut, Laurent Bidault, Joëlle Amédée, Didier Letourneur, Jean-Christophe Fricain, Mathilde Fenelon

**Affiliations:** ^1^ Université de Bordeaux, INSERM 1026, Bordeaux, France; ^2^ SILTISS, Saint-Viance, France; ^3^ Université Paris Cité, Université Sorbonne Paris Nord, INSERM 1148, LVTS, Hôpital X Bichat, Paris, France; ^4^ CHU de Bordeaux, Service de Chirurgie Orale, Bordeaux, France; ^5^ Centre de Compétence des Maladies Rares, Orales et Dentaires, Pôle d'Odontologie et Santé Buccale, Centre Hospitalier Universitaire Bordeaux, Bordeaux, France

**Keywords:** preclinical model, micro-computed tomography, guided bone regeneration, membrane, bone graft substitute

## Abstract

Innovative biomaterials are increasingly being investigated for guided bone regeneration (GBR) in oral and maxillofacial surgery. However, the development of relevant preclinical models still need to be consiedered. This study aimed to propose a standardized and reproducible maxillary bone defect model in rats that could be relevant to evaluate new materials for GBR. Three defect sizes in rat maxillary of 2.8, 3.3, and 4.5 mm in diameter were compared. Bone formation was followed until 12 weeks post-surgery using longitudinal micro-computed tomography and histological analysis. The defect was subsequently filled by an osteoconductive bone substitute (GLYCOBONE), then covered either by a new natural polysaccharide membrane supplemented with hydroxyapatite, or by a commercial collagen membrane (BIO-GIDE). Results showed little spontaneous tissue regeneration for empty defects (bone volume fractions (BVF) below 40% after 12 weeks). The smallest size defect (2.8 mm) was the most reproducible and was thus selected for testing GBR membranes. Defects filled with GLYCOBONE and covered with membranes displayed for both materials accelerated and substantial bone regeneration (with BVF that reached 80% after 12 weeks). Histological sections showed immature bone formation for the empty defects, whereas the defects filled with the GBR membranes highlighted a lamellar structured bone. The polysaccharide membrane was as effective as the commercial collagen membrane to guide bone tissue regeneration. This study provides a step-by-step protocol of a new standardized rat maxillary bone defect model. In line with ethical and financial considerations, this rodent model should be considered as a preliminary level before performing larger animal experiments.

## 1 Introduction

Oral and maxillofacial bone defects can arise from various causes such as tumor resection, severe trauma, congenital malformation, bone resorption due to periodontal disease, or tooth loss (Dimitriou et al., 2011; Lanza et al., 2014; Mansour et al., 2020; Fenelon et al., 2023). The management of such defects is a public health issue, as they can lead to severe oral dysfunction and facial deformities resulting in impaired quality of life. It requires bone reconstruction procedures to allow subsequent rehabilitation by placement of dental implants (Chiapasco et al., 2006). Guided bone regeneration (GBR) is one of the most commonly used reconstruction techniques for jaw bone augmentation (Abtahi et al., 2023). However, these surgical procedures still remain a clinical challenge for oral and maxillofacial surgeons mainly due to the limitations of autologous bone grafts (*i.e.*, the limited availability and additional donor site morbidity) (Baj et al., 2016). Bone tissue engineering (BTE) strategies have thus gained increasing interest for the development of innovative biomaterials to regenerate alveolar bone (Gómez et al., 2016; Wubneh et al., 2018). For their management in clinical needs, these biomaterials must meet specific criteria including biocompatibility, biodegradability, osteoinductive or osteoconductive properties, mechanical strength, and ease of handling suitability (Sanz et al., 2019).

Preclinical assessment of novel biomaterials using clinically relevant animal models is essential for translational research (Shanbhag et al., 2017). While large animal models are often valued for their anatomical and functional similarities to humans (such as comparable size, load-bearing capacity, and masticatory forces) (Histi et al., 2011; Gomez et al., 2021; Unnikrishnan et al., 2022; Vdoviaková et al., 2023; Fricain et al., 2018)), their use raises important ethical considerations and should be limited to later stages of development (Guide for the Care and, 1996; Burden et al., 2015; Zeiter et al., 2020). In contrast, small animal models, particularly rats, offer several scientific and practical advantages for early-stage evaluation of bone regeneration strategies. These include their well-characterized genetics, the availability of standardized surgical protocols, and the ability to perform high-resolution longitudinal imaging (e.g., *in vivo* micro-CT) to monitor healing dynamics. Rats also exhibit intramembranous ossification in craniofacial bones, similar to human alveolar bone, making them suitable for modeling periodontal and maxillofacial defects. Additionally, the use of small animals can facilitate the investigation of biological variables, such as sex-related differences in bone healing, which are more logistically challenging in large models (Chen et al., 2025). Together, these factors make the rat an appropriate and valuable model for the initial screening and optimization of bone regenerative therapies prior to progression to large animal or clinical studies.

For this purpose, it is crucial to select relevant small animal models depending on the targeted applications. In oral and maxillofacial surgery, rodents mainly offer two well-established models for investigating bone regeneration: calvarial and mandibular bone defects (Kim and Kim, 2013; Hatt et al., 2022). Craniofacial bones are formed through intramembraneous ossification where cells differentiate into osteoblasts to deposit osteoid matrix leading to calcification (Scholz et al., 2019). Calvarial defects are the most commonly employed models due to their reproducibility and the simplicity of subsequent radiological and histological analyses (Spicer et al., 2012; Fénelon et al., 2018). Calvaria bone is derived from the mesoderm and cells highlight a weaker osteogenic potential compared to bones derived from the neural crest (e.g., mandible, maxillae) (Galea et al., 2021). In addition, these defects are not suitable for investigating load-bearing capacity. To address this limitation, rat mandibular bone defects have been performed to test biomaterials for oral applications (Zhao et al., 2023; Liu et al., 2019; Ma et al., 2021; Taguchi and Lopez, 2021). However, surgical interventions in this area present major difficulties such as the risk of masseter injury or damage to crucial anatomical structures such as the parotid duct, the facial nerve and artery or dental roots (Liu et al., 2019; Padial-Molina et al., 2015). Liu et al. also emphasized that these studies usually lack standard and reproducible criteria such as age and gender of the animals, defect location and size, as well as operative procedures and observation time (Liu et al., 2019). Additionally, these defects are all performed in the mandibular ramus instead of alveolar bone which is usually the targeted location for bone regeneration in clinical practice. Indeed, the mandibular ramus is anatomically distant from dental roots and the periodontal ligament. These two structures can influence locally the microenvironment to mimick bone healing in humas. Therefore, a maxillary alveolar bone defect model could be considered as a suitable alternative for the two models mentioned above. To our knowledge, there is no consensus concerning a well-established and characterized maxillary bone defect model of critical size in rodents to assess biomaterials for oral and maxillofacial surgery (Liu et al., 2019; Nguyen et al., 2009; Toyota et al., 2021; Liu et al., 2022; Nie et al., 2019; Wheelis et al., 2021; Raposo-Amaral et al., 2014).

Therefore, the aim of this study was to develop a standardized and reliably reproducible bone defect model in the rat maxilla that will be useful to further assess biomaterials in the field of oral and maxillofacial surgery. For this purpose, three different sizes of maxillary bone defects were first assessed (2.8, 3.3, and 4.5 mm). Then, a bone substitute (GLYCOBONE) already identified as osteoconductive material (Fricain et al., 2018; Schlaubitz et al., 2014; Maurel et al., 2021; Ribot et al., 2017), and covered by either a new GBR membrane under development based on biodegradable polysaccharides (Ahmed et al., 2023), or a commercial collagen membrane (BIO-GIDE, Geistlich, Switzerland), were subsequently implanted in the selected model to validate its suitability for testing materials for oral and maxillofacial applications.

## 2 Methods

### 2.1 Material preparation

Three materials were used in this study: a bone substitute (GLYCOBONE), a polysaccharide-based membrane containing hydroxyapatite particles, and a commercial and widely-used collagen membrane (BIO-GIDE, Geistlich, Switzerland).

#### 2.1.1 Bone substitute preparation

The bone graft substitute (GLYCOBONE) was composed of a polysaccharidic blend (pullulan/dextran with a ratio of 75:25) combined with hydroxyapatite. Previous studies already investigated its osteoconductive properties (Fricain et al., 2018; Schlaubitz et al., 2014; Maurel et al., 2021; Ribot et al., 2017; Fricain et al., 2013; Frasca et al., 2017). Microbeads of gamma-irradiated GLYCOBONE were rehydrated (Ahmed et al., 2023) with a sterile NaCl solution (0.9% (m/v)) before their implantation in the maxillae.

#### 2.1.2 Polysaccharide-based membrane preparation

The pullulan/dextran-based membrane containing hydroxyapatite (GlycoMembrane) was prepared according to a previously reported procedure (Ahmed et al., 2023). Membrane was cut into 5 × 5 mm pieces to cover the bone defect and was stored at room temperature before implantation.

#### 2.1.3 Collagen membrane preparation

The resorbable collagen membrane (BIO-GIDE) was cut into 5 × 5 mm pieces and stored at room temperature before implantation.

### 2.2 Animal experiments

Two rat experimental procedures were performed in this study. The first one aimed to develop a standardized defect in the maxilla by assessing three different bone size defects. The surgical site, the alveolar ridge between the maxillary incisor and palatal suture, was chosen as it reproduced: 1) a tooth-adjacent bone loss pattern, 2) the influence of mechanical forces (mastication, occlusion), 3) a functionally active site comparable to anterior alveolar defects in humans, and 4) the possibility to evaluate GBR strategies (*i.e.,* with membrane coverage and bone substitutes), which are widely used in periodontal contexts. The size of the defect was chosen accordingly to fit the surgical site as defects above 5 mm could not be drilled in this region. The second experimentation focused on the validation of this model by testing BTE materials. Both animal procedures were performed following the principles of Laboratory Animal Care formulated by the National Society for Medical Research and complied with the ARRIVE guidelines. They were approved by the Animal Care and Experiment Committee of the University of Bordeaux, Bordeaux, France. Experiments were conducted in accredited animal facilities following European recommendations for laboratory animal care (directive 86/609 CEE of 24/11/86). Prior to the surgeries, animals were quarantined and housed in cages for acclimatization during 7 days. All surgeries were performed by one trained surgeon in aseptic conditions with sterile surgical materials. The surgeon has trained on eight rats before the surgery to enable reproducibility between defects and visualize the maximum defect sized suited in this anatomical part. The accurate position of the defects was evaluated through the micro-computed tomography (micro-CT) images.

### 2.3 Development of a new maxillary bone defect model

**FIGURE 1:**
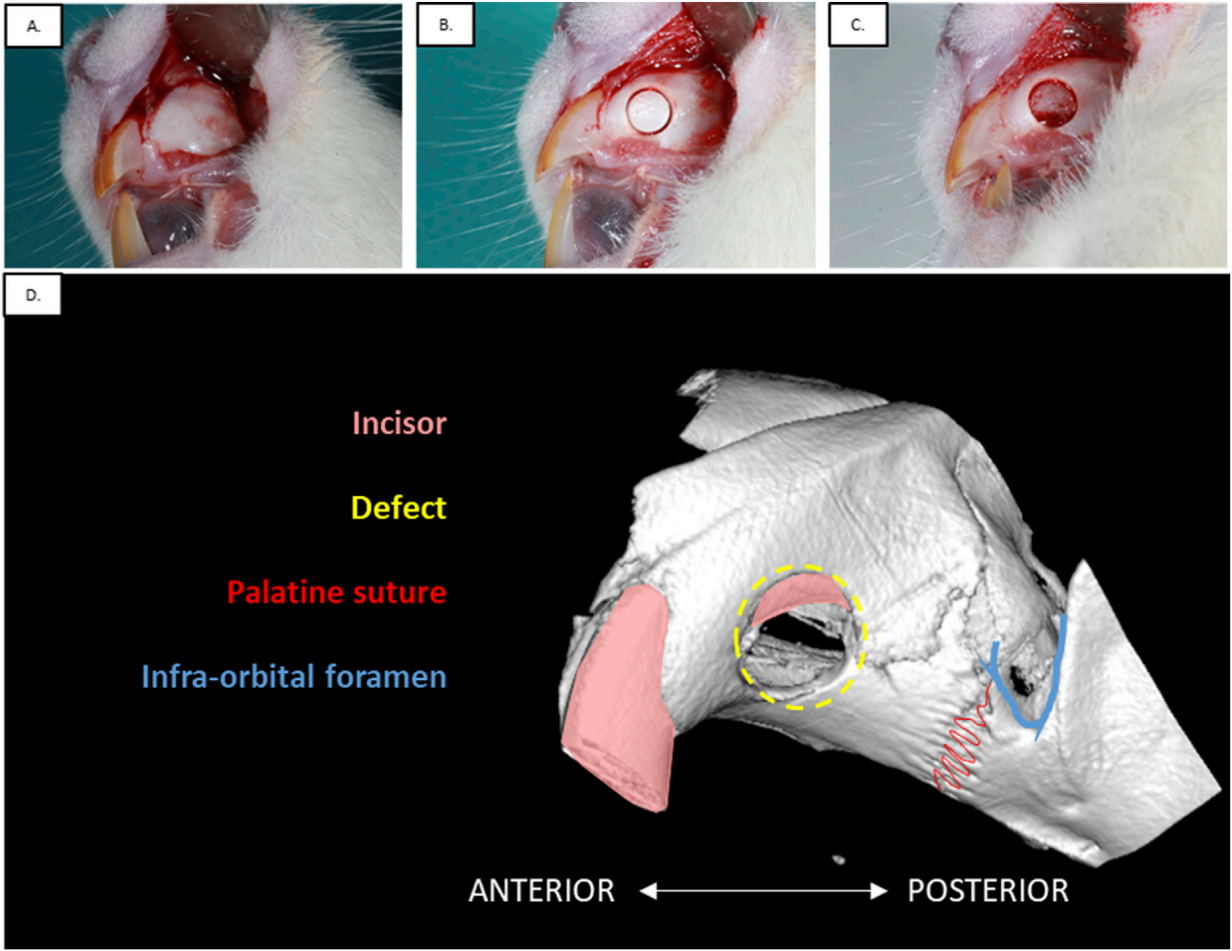
Experimental procedure of the alveolar defect surgery in rat maxillary. **(A)** Incision of the gum was performed to raise a full-thickness flap and expose the bone. **(B)** Bone defect was created using the appropriate size trephine burr: 2.8 mm, 3.3 mm or 4.5 mm (Defect size of the present picture: 3.3 mm). **(C)** Alveolar bone was removed and hemostasis was performed. **(D)** Anatomical features of the maxilla in rat.

Twelve adult male Sprague-Dawley rats (11-week-old, 350–400 g body weight) were used for the first study (approval by the French Ethics Committee under APAFIS n° 202002061614957v4). To limit pain, buprenorphine (0.05 mg/kg) was injected intraperitoneally before the surgery. Animals were anesthetized with a mixture of ketamine (50 mg/kg) and xylazine (10 mg/kg) diluted in physiological serum (NaCl 0.9% (m/v)) administrated by intraperitoneal injection. A 1 cm ridge incision was performed and a full-thickness flap was subsequently raised to expose the maxillary bone. Animals were divided into three groups composed of four animals with three different defect sizes: 1) 2.8 mm, 2) 3.3 mm, and 3) 4.5 mm diameter (*n* = 8 defects per condition). Under irrigation, a defect was then created using a different trephine bur (Helmut Zepf, Germany) for each of the three diameters. The defect was performed on the buccal bone in the middle of the maxilla between the palatal suture and the beginning of the crown incisor at least 2 mm away from the incisor tooth (Figure 1). Hemostasis was performed by local compression. Defects were created on each maxilla, resulting in two defects per animal. Mucosa flaps were then sutured using a 6/0 Prolene (Ethicon, France) suture thread. Surgery lasted from 30 to 40 min per animal. To facilitate animal recovery, softened food was placed inside the cages and intraperitoneal injections of buprenorphine (0.05 mg/kg) were administered daily for 48 h. After 12 weeks, animals were sacrificed (*n* = 8 maxillae per condition) by carbon dioxide under general anesthesia (isoflurane 2% (v/v)). Maxillae were collected and fixed overnight in PFA 4% (ANTIGENFIX, Diapath, France) at 4°C.

### 2.4 Assessment of the proposed maxillary bone defect model for material testing

The second experimentation was also performed on twelve adult rats, 11 weeks old (agreement APAFIS n° 35311-2022020911368338v2) and aimed to validate the above model to implant and assess new BTE materials for bone regeneration. Therefore, the same procedure was performed. Briefly, after anesthesia, a 2.8 mm in diameter defect was performed under irrigation using a trephine bur (Helmut Zepf, Germany) on each rat maxilla. To mimic clinical GBR procedures, two different conditions were allocated to each animal: 1) defect filled with the bone graft substitute (GLYCOBONE) and subsequently covered by a collagen membrane (BIO-GIDE), or 2) defect filled with the bone graft substitute (GLYCOBONE), then covered by the polysaccharide membrane (GlycoMembrane), (*n* = 12 maxillae per condition). *In vivo* micro-computed tomography was performed for up to 12 weeks to investigate bone regeneration. At the last timepoint, animals were sacrificed by intracardiac injection of sodium pentobarbital (200 mg/kg). Maxillae were collected and fixed overnight in PFA 4% (ANTIGENFIX, Diapath, France) at 4°C.

### 2.5 *In vivo* radiographic assessment of the rat maxillae

#### 2.5.1 *In vivo* longitudinal scanning

*In vivo* micro-computed tomography (micro-CT) (SKYSCAN 1276, Bruker, United-States) was performed for up to 12 weeks to follow longitudinally the bone defect regeneration. Under general anesthesia (isoflurane 2%–3% (v/v) in air/oxygen), rats were placed in decubitus in the scan stage. All micro-CT scans were conducted at an accelerating voltage of 80 kV with a current of 200 µA to obtain a resolution of 42 µm using an Al + Cu filter (Orhan et al., 2020). Micro-CT acquisitions were performed the day after the surgery (T0), and then after 2, 4, 6, 8, and 12 weeks post-operatively. After image acquisition, the data were reconstructed with the NRECON software (version 1.7.1, Bruker, United-States). Correction for post-alignment was optimized per scan if needed. Smoothing level, ring-artifact reduction, and beam hardening were applied with 1%, 4%, and 20% values, respectively (Chatterjee et al., 2017). The dynamic image range of the histogram was set from −1000 to 17000 HU. Files were exported to DICOM format for radiographic analysis.

#### 2.5.2 Micro-CT analysis

The cross-sectional slices were visualized using the VGSTUDIO MAX software (version 2022.3, Volume Graphics, Germany). First, 3D images obtained from the T0 timepoint were used to face the defect on the sagittal plane. A cylindrical volume of interest (VOI) was drawn according to the three different tested diameters (2.8 mm, 3.3 mm, and 4.5 mm) and with 330-μm in depth to fit the initial surgical defect. Due to the longitudinal follow-up for the same defect, the orientation of the previous timepoint was used to face the defect using the “Best fit registration” tool on VGSTUDIO MAX software. Then, bone volume fraction (BVF) was computed for each timepoint (T0, 2, 4, 6, 8, and 12 weeks) from the extracted VOI. The BVF refers to the ratio of the segmented bone volume (BV) to the total volume (TV) within the defined VOI (Bouxsein et al., 2010). In some defects, the incisor root interfered with the VOI. To avoid over-estimation of BVF, the incisor root was segmented using the growing seeded tool brush to erase its signal. Each scan was reconstructed using the same calibration system to distinguish bone and background.

### 2.6 Histological analysis

Maxillae collected 12 weeks after the surgery were decalcified with EDTA-based MICRODEC (Diapath, France) for 4 weeks, under gentle agitation. Maxillae were cut in half to obtain hemi-maxillae. Samples were then dehydrated in increasing ethanol baths and processed for embedding in paraffin. Coronal cuts of rat hemi-maxillae were made to obtain 7-μm-thick serial sections in the middle of the defect. Masson-Goldner’s trichrome staining was used to visualize bone formation at the defect site (Kiernan, 2015). Briefly, sections were stained with Mayer’s hemalum solution (Sigma, United-States) for 4 min and rinsed in tap water. After, they were placed in ponceau S 0.2%/fuchsin acid 0.1% solution for 3 min. Differentiation of the tissues was obtained using orange G 2%/phosphomolybdic acid 5% solution for 1 min 30 s. Slides were finally stained in light green 1% solution for 6 min.

Picrosirius red staining was also performed to further observe collagen fibers’ orientation. Brightfield images were obtained using a slide scanner (Nanozoomer 2.0, Hamamatsu Photonics, France). Sections stained with picrosirius red were observed under crossed polarizing filters on a Nikon microscope (Eclipse 80i, Nikon, Japan). This method, relying on the birefringence of collagen fibers, is used in combination with polarized light microscopy to specifically highlight collagen networks (Liu et al., 2021; Rittié, 2017).

### 2.7 Statistical analysis

Results were expressed as mean ± SD and “*n*” indicated the number of defects tested. The GraphPad Prism Software 8.2.0. (La Jolla/CA, United States) was used to perform statistical analysis. We first performed a normality test using a D’Agostino and Pearson omnibus normality test. Differences for independent samples were evaluated with the non-parametric Kruskal–Wallis test and Dunn’s multiple comparison test. The non-parametric Mann-Whitney test (two-tailed) was used to compare two groups. Differences were considered significant and indicated with * when *p* < 0.05 (*), *p* < 0.01 (**), *p* < 0.001 (***), and *p* < 0.0001 (****).

## 3 Experiments

### 3.1 Validation of a new standardized defect model in rat maxillary

#### 3.1.1 Surgical evaluation

The different steps of the surgical procedure are described in Figures 1A–C. The anatomical features composing the maxillary bone are visible in Figure 1D. Three different defect sizes were performed on the maxillary bone at 2.8 mm, 3.3 mm, and 4.5 mm. All animals (*n* = 12) tolerated well the surgery, whether the defect was left empty or filled with the materials. They did not show any wound complication post-operatively. No infection was detected at any time after surgery, nor masticatory dysfunction.

To ensure reproducibility, the defect was positioned antero-posteriorly between the incisor crown and the palatine suture. Depending on the trephine bur size, accessibility to the maxilla was challenging. Therefore, the smallest size defect of 2.8 mm in diameter was the easiest to perform, showing the most reproducible location.

#### 3.1.2 Micro-CT analysis of mineralized newly formed tissue within the three different size bone defect models

**FIGURE 2:**
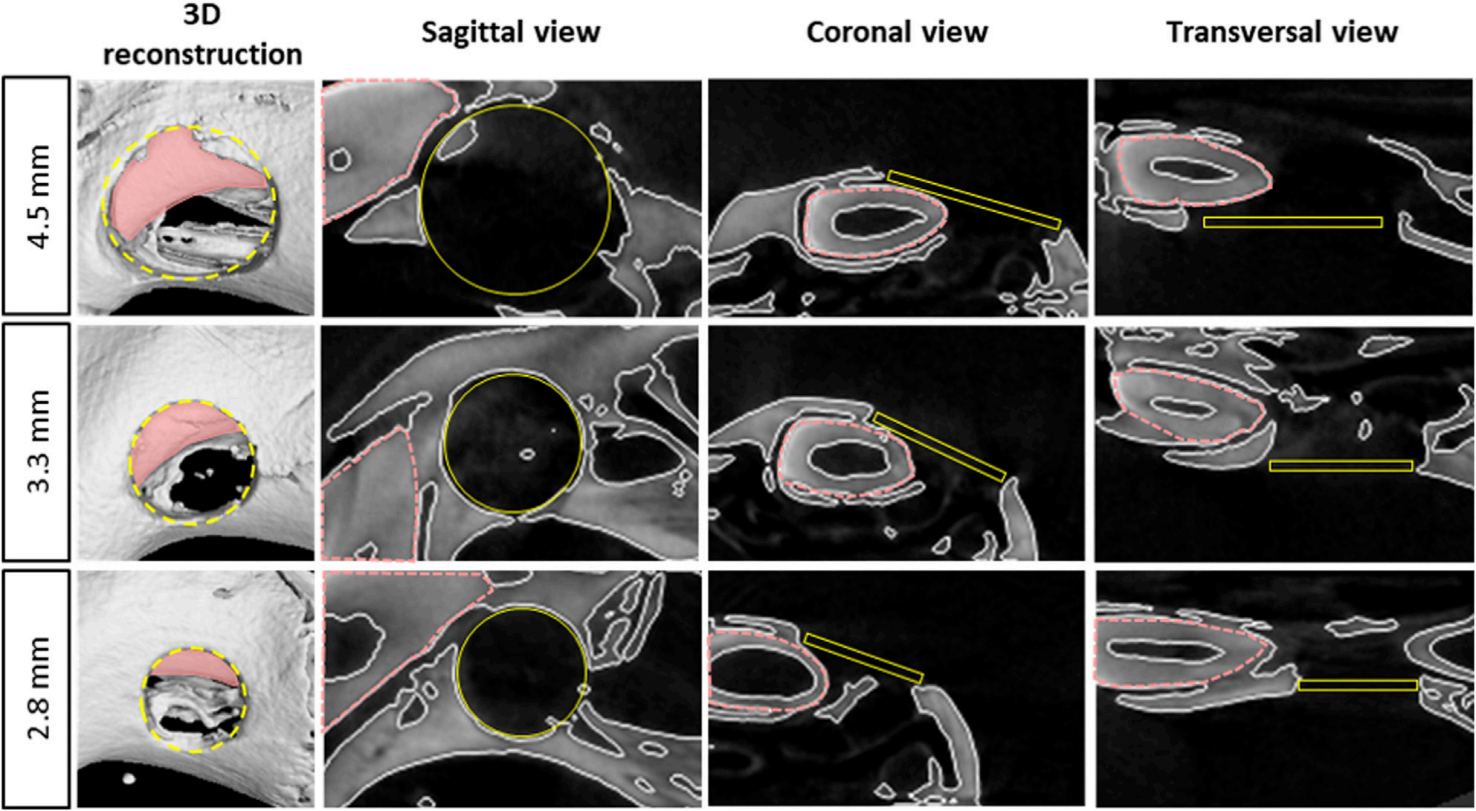
Radiological representation of the different size bone defects in the maxillary bone - 3D reconstruction and 2D views of the different size defects on rat’s maxilla at the middle of the defect using micro-CT images, pink doted lines represent the incisor, yellow circles and rectangles represent the computed volume of interest (VOI) for micro-CT analysis.

Representative 3D reconstructions and 2D planes of the three defect size models, as well as the volume of interest (VOI) location to fit the defect, are shown in Figure 2. As observed in coronal sections, VOI position may include the incisor root, thereby requiring incisor segmentation for further quantification of the bone volume fraction (BVF).

**FIGURE 3:**
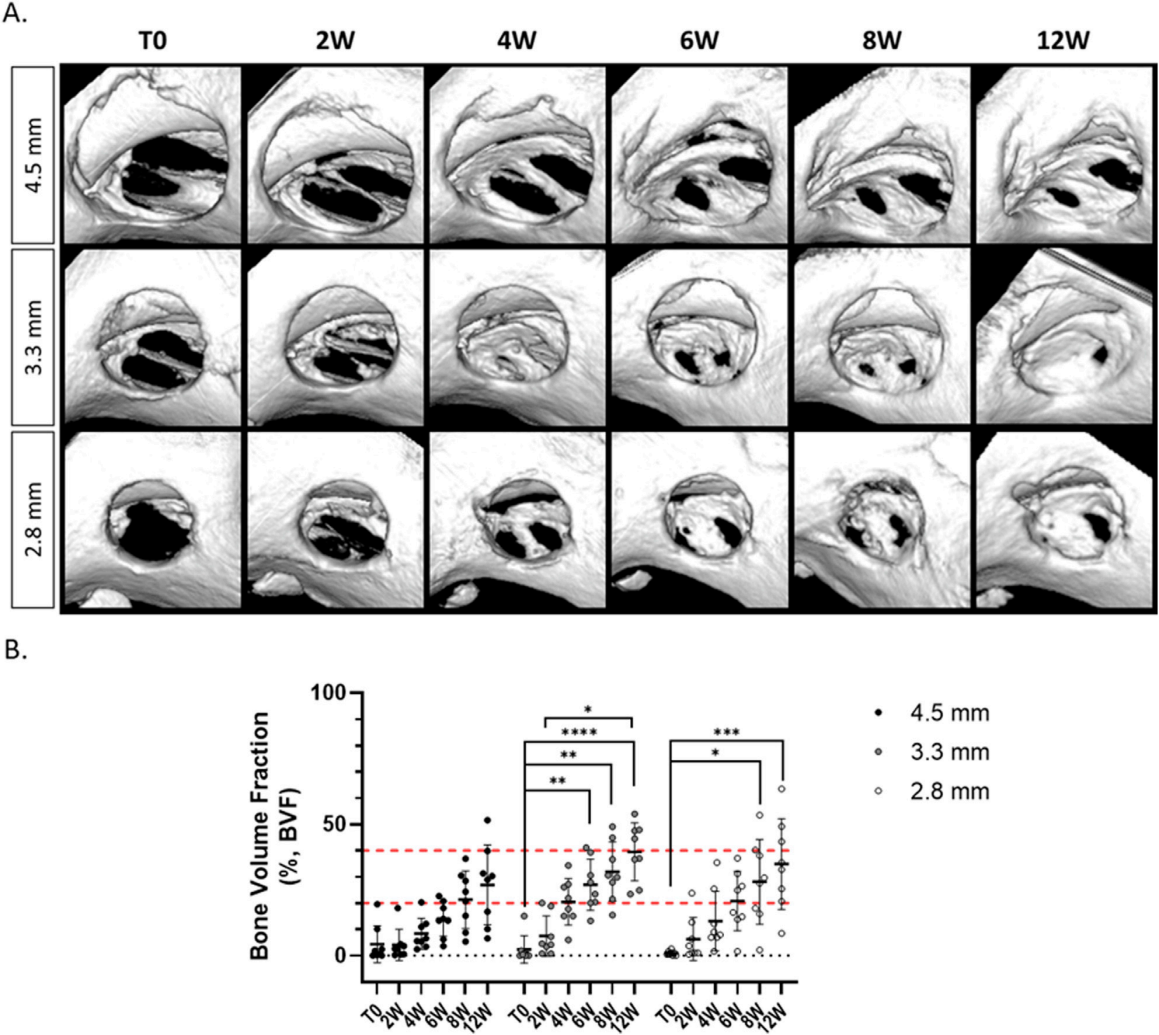
Longitudinal micro-CT analysis of alveolar bone defect regeneration, left empty according to the three defect sizes. **(A)** 3D reconstruction of the defects at different timepoints. **(B)** Micro-CT analysis of the Bone Volume Fraction at different timepoints (*n* = 8/condition), non-parametric Kruskal–Wallis test compared within the same defect size, *p < 0.05, **p < 0.01, ***p < 0.001, ****p < 0.0001.

Longitudinal follow-up of the defects was investigated until 12 weeks post-surgery (Figure 3). Few mineralized spots were observed within the empty defects up to 4 weeks, whatever the defect size (with a BVF lower than 20%). BVF analysis highlighted a slow regeneration rate from 6 to 12 weeks, without significant enhancement overtime, never reaching more than 40% of the total VOI (at 6W BVF [%]: 4.5 mm = 13.99 ± 6.64%; 3.3 mm = 26.98 ± 9.71%; 2.8 mm = 20.77 ± 11.30%, and at 12W BVF [%]: 4.5 mm = 26.92 ± 15.16%; 3.3 mm = 39.48 ± 11.07%; 2.8 mm = 34.85 ± 17.29%). Incomplete mineralization was observed until the last timepoint regardless of the defect diameters.

Additionally, we observed that the defect of 2.8 mm diameter was the most reliable and reproducible for the surgeon since the defect was not to close from the crown of the incisor or from the palatine suture. Larger defects showed to be mispositioned (*e.g.,* if the defect was too close from the incisor or the palatine suture, resorption of the defect happened), and were often oversized (data not shown).

#### 3.1.3 Histological analysis of newly formed tissue within the three different size bone defects in the maxillary rat model

**FIGURE 4:**
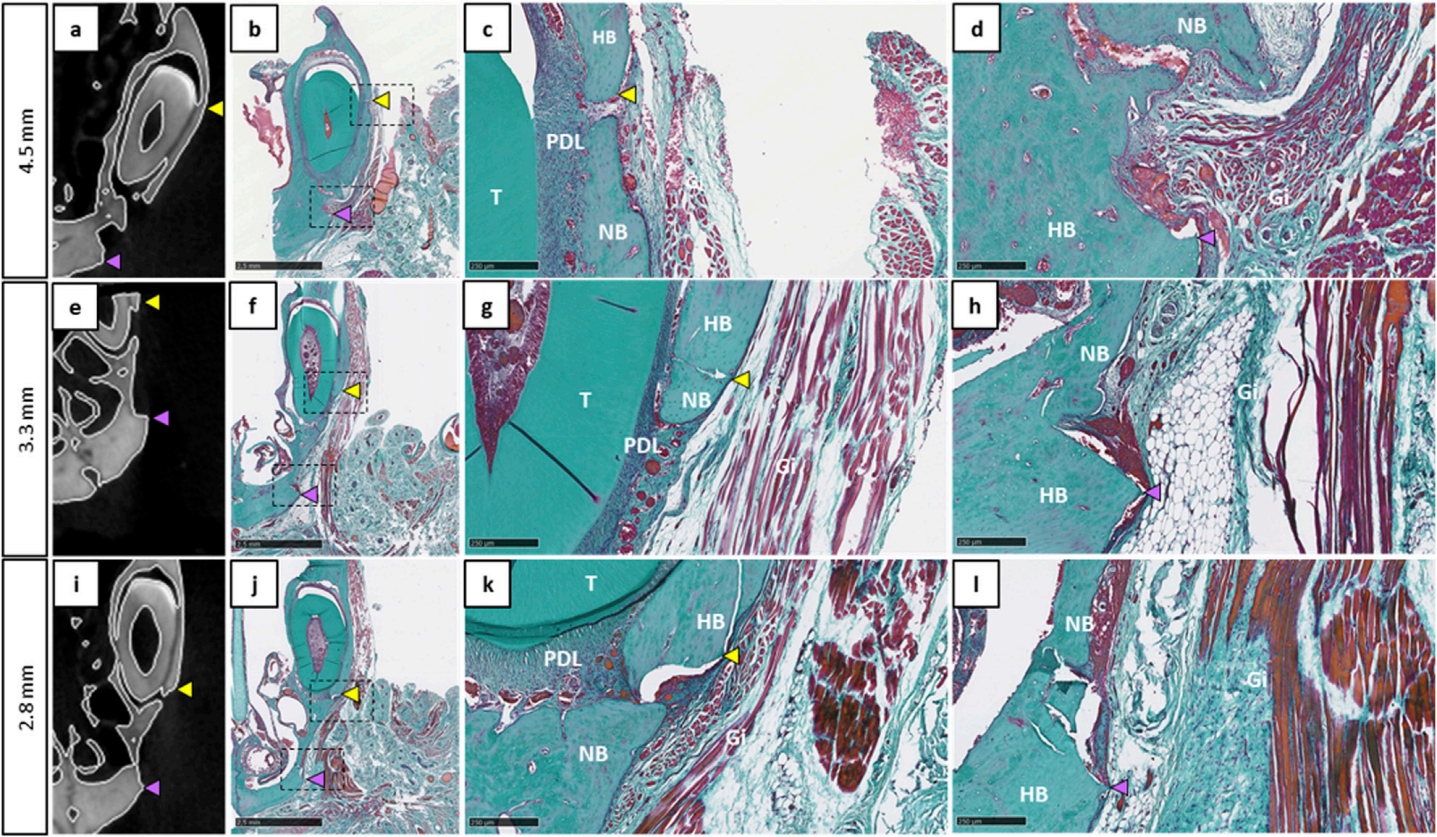
Histological evaluation of the different bone defect sizes left empty at 12 weeks post-operation. **(a,e,i)**: Coronal 2D images showing approximately the middle of the defect 12W after surgery. **(b,f,j)**: Corresponding histological sections stained by Masson-Goldner’s trichrome (scale bar: 2.5 mm). **(c, d, g, h, k, l)**: Magnification view at the edges of the defect (scale bar: 250 µm), yellow arrowheads represent the upper defect edge, purple arrowheads represent the lower defect edge, Gi: gingiva, HB: host bone, NB: new bone, PDL: periodontal ligament, T: tooth.

Qualitative analysis of histological staining at the end of the experiment (12 weeks) for the different size bone defects left empty at the beginning of the surgery was consistent with the radiological results (Figure 4). The three conditions (*i.e.*, the three tested size defects) showed the absence of complete bone regeneration and revealed only an immature newly bone tissue. The defect was mainly filled by a disorganized bone and connective tissue. The edges of the defects were sometimes difficult to distinguish due to bone thinning, especially with the 4.5 mm diameter for which clear delimitation of the upper edges was challenging.

Finally, the maxillary bone defect of 2.8 mm diameter was the easiest to perform during the surgery, allowing reproducible radiological and histological analysis. Consequently, it was selected for testing biomaterials in further analysis.

### 3.2 Assessment of bone regeneration after BTE materials implantation within the bone defect in the maxillary rat model

#### 3.2.1 Micro-CT analysis of bone regeneration after GBR procedure

BTE materials were then implanted in 12 rats with the previously selected maxillary bone defect model of 2.8 mm diameter to validate its suitability to study various types of materials for oral bone regenerative applications. GBR procedure was performed by filling the defects with the bone substitute (GLYCOBONE) already known for its osteoconductive properties, and covered either by a commercially available and widely used collagen membrane (BIO-GIDE), or by a new pullulan/dextran-based membrane containing hydroxyapatite (GlycoMembrane).

**FIGURE 5:**
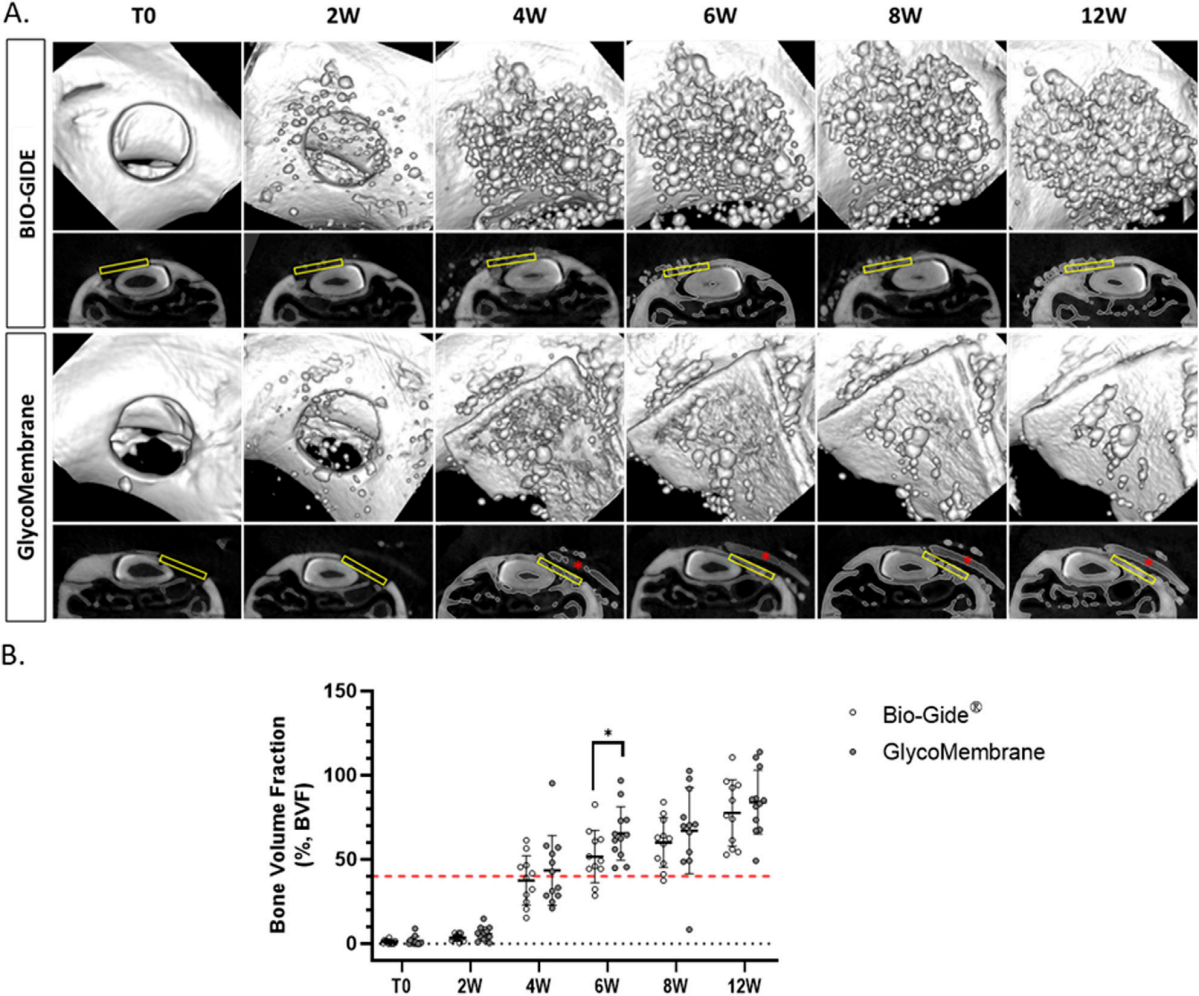
Longitudinal micro-CT analysis of alveolar bone defect after GBR procedure. **(A)** 3D reconstruction and 2D coronal sections of the defects filled with GLYCOBONE and covered by a membrane (BIO-GIDE or GlycoMembrane) at the different timepoints. The yellow rectangles represent the region of interest (ROI) and the red asterisks represent the residual membrane. **(B)** Micro-CT analysis of the Bone Volume Fraction at different timepoints (*n* = 11 for BIO-GIDE and *n* = 12 for GlycoMembrane), non-parametric Mann-Whitney test, *p < 0.05.

The implanted GLYCOBONE bone substitute is known for its initial radiolucent properties (Fricain et al., 2018), thereby allowing to follow its progressive mineralization, as demonstrated in the 3D reconstructed images (Figure 5). Since GlycoMembrane shared similar composition with the microbeads, mineralization of the membrane could also be monitored, as the membrane was clearly distinguishable from the microbeads. Membrane mineralization started to be observed at 4 weeks after the surgery. Bone regeneration was similar, regardless of the membrane used to cover the bone substitute for up to 4 weeks. In contrast, bone regeneration was significantly greater 6 weeks after the surgery when the bone substitute was covered by the GlycoMembrane compared to the commercial membrane BIO-GIDE (at 6W BVF [%]: BIO-GIDE = 51.71 ± 15.55% and GlycoMembrane = 65.43 ± 15.08%, *p* < 0.05). Finally, a gradual bone regeneration was observed in both conditions until the late timepoint (at 12W BVF [%]: BIO-GIDE = 77.63 ± 19.69% and GlycoMembrane = 84.08 ± 18.90%). Micro-CT analysis also showed that both membranes successfully maintained the filling material inside the bone defect. However, some variability was observed on the Micro-CT analysis as for some defects, the membranes did not perfectly cover the defect (histological slices, data not shown) and some materials may have leak from the defect.

#### 3.2.2 Histological analysis of the maxillary rat model after GBR procedure

**FIGURE 6:**
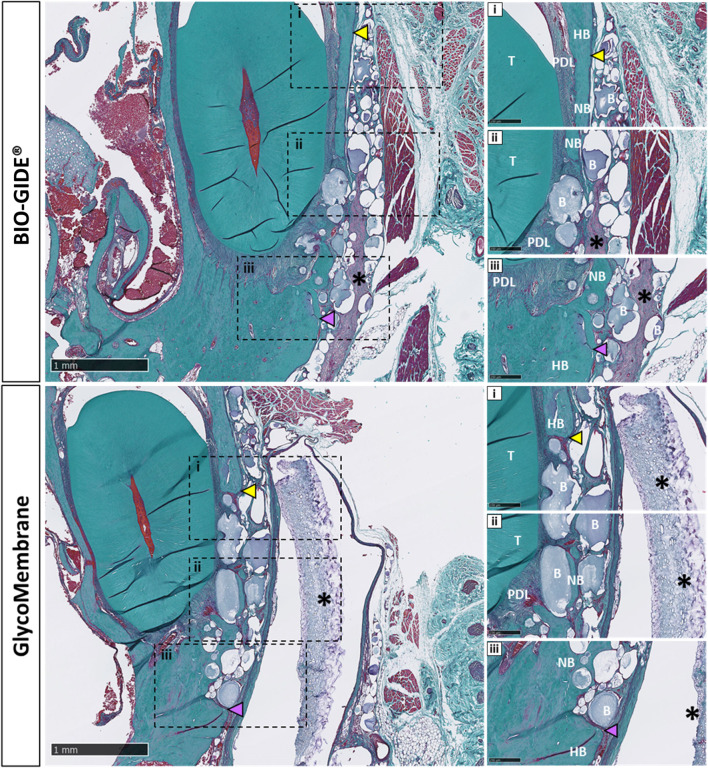
Histological evaluation of the different bone defect filled with GLYCOBONE and covered by a membrane (BIO-GIDE or GlycoMembrane), showing consistent bone formation inside the defect and around the micro-beads after 12 weeks (scale bar: 1 mm). i, ii, iii: Higher magnification view at the edges of the defect (scale bar: 250 µm). Black asterisks represent remaining membrane, yellow arrowheads represent the upper defect edge, purple arrowheads represent the lower defect edge, B: microbeads, Gi: gingiva, HB: host bone, NB: new bone, PDL: periodontal ligament, T: tooth.

Histological sections of the 2.8 mm defects filled with GLYCOBONE and covered either by GlycoMembrane, or by the collagen membrane (BIO-GIDE) were stained by Masson-Goldner’s trichrome 12 weeks after the surgery (Figure 6). Defects were mainly filled by the microbeads which showed integration into the host tissue and were in close contact with native and newly formed bone. A well-organized lamellar bone was thus observed around microbeads. Both membranes were integrated into the surrounding tissue, keeping microbeads inside the defect. The collagenous membrane BIO-GIDE was colonized by the surrounding cells overtime, leading to its resorption. Interestingly, the polysaccharide membrane (GlycoMembrane) maintained its barrier function for longer, with no cellular infiltration observed for up to 12 weeks. A thin fibrotic capsule was observed around GlycoMembrane. However, no inflammatory cells were observed around the membrane as previously reported (Ahmed et al., 2023). For both membrane conditions, new bone formation was observed after 12 weeks in direct contact with the microbeads.

**FIGURE 7:**
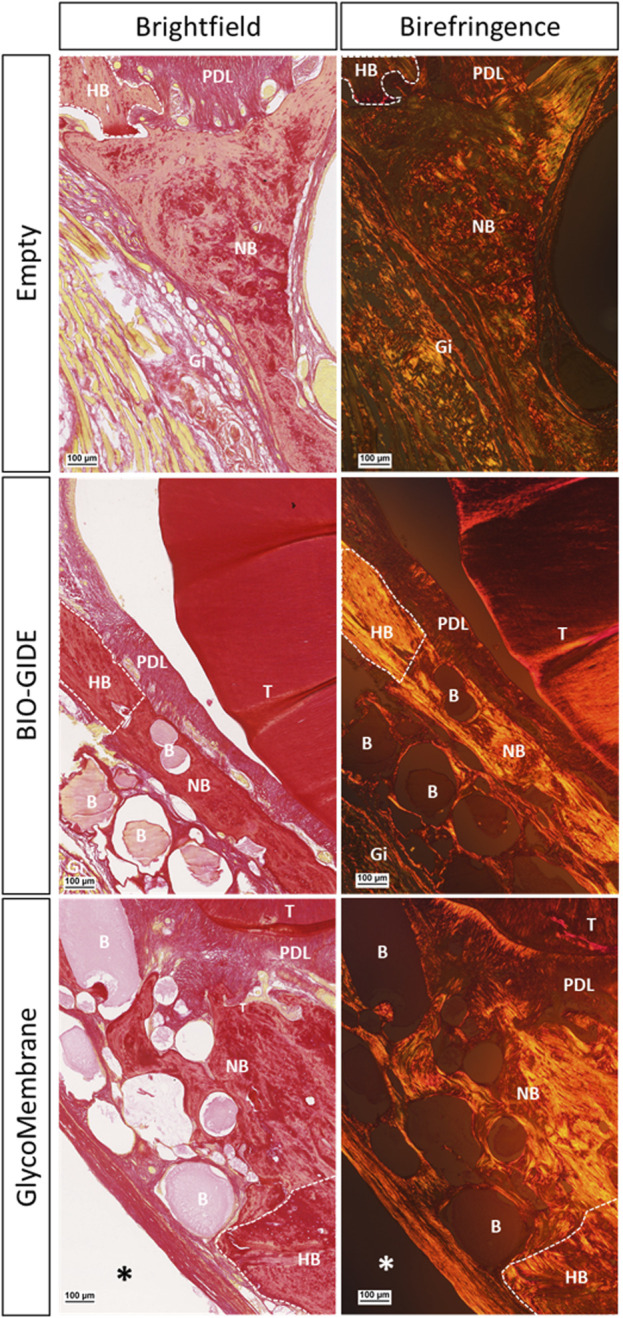
Picrosirius red staining to evidence collagen fibers orientation inside the alveolar defects left empty or covered with a membrane (BIO-GIDE or GlycoMembrane) at 12W post-surgery (scale bar: 100 µm). Histological sections under brightfield view and polarized light view (birefringence and grayscale modes) of the slides for defect left empty or covered by a membrane. Black and white asterisks represent remaining membrane, white dotted lines represent defect edges, B: microbeads, Gi: gingiva, HB: host bone, NB: new bone, PDL: periodontal ligament, T: tooth.

Tissue sections were also stained using picrosirius red to evidence collagen fiber orientation around the tested materials compared to the empty defect (2.8 mm) (Figure 7). Qualitative analysis under polarized light showed woven bone with unorganized collagen fibers inside the empty defect area, whereas osteoid tissue formation and lamellar tissue with parallel organized collagen fibers were evidenced in the bone defects filled using GBR procedure.

## 4 Discussion

This study aimed to develop a standardized maxillary bone defect in rats and validate this model to study BTE materials. The first part of the study focused on the step-by-step description of the experimental model, and intended to determine the most appropriate size and time for the bone defect for bone reconstruction. The second part highlighted the successful application of this model to evaluate innovative biomaterials to regenerate bone in oral and maxillofacial surgery.

The first goal was to develop a standardized maxillary bone defect model. To this end, three different size defects (*i.e.*, diameter of 2.8, 3.3, and 4.5 mm) were analyzed comparing the surgical feasibility and bone regeneration. Maxilla was chosen as it offers easy access to the alveolar bone without damaging surrounding tissues and provides sufficient bone volume to test biomaterials. At the same time, this area was the most relevant to investigate alveolar bone regeneration with its proximity to incisor root. To facilitate the surgical technique and ensure optimum shape reproducibility, a circular defect was chosen. Firstly, radiographic analysis showed that the three defects demonstrated incomplete mineralization but could not be consider as critical size defects. The term “critical size defect” can refer to the smallest defect size that cannot spontaneously heal (Spicer et al., 2012). However, there is often a lack of information regarding animal models specifically designed to study critical size defects (McGovern et al., 2018). This is primarily due to the fact that evaluating the smallest size defect is not consistently performed, and experimental endpoints typically do not extend enough time (Spicer et al., 2012). In this study, the BVF measured at the late timepoint (*i.e.*, 12 weeks) did not reach more than 40% of regeneration and complete healing was not observed in any of the conditions. Furthermore, bone repair did not further increase significantly after 6 weeks after the surgery. It was also observed that the defect of 2.8 mm of diameter was easier to perform and to position correctly between the crown incisor and the palatine suture with the smallest trephine bur. Consequently, this smallest defect size (2.8 mm diameter) was selected.

We then assessed this model as a reliable and reproducible way to investigate innovative bone repair materials for oral surgery. For now, large animal models such as pig or sheep are usually performed to test such devices for their similar physiological features and translation to human needs (Histi et al., 2011; Gomez et al., 2021; Fricain et al., 2018; Hatt et al., 2022). For early-stage investigations, the feasibility of such models can be challenging for researchers. Also in accordance with the 3Rs principles, which request avoiding large animal models when a small animal can fulfill the research objectives (Guide for the Care and, 1996; Burden et al., 2015), diverse types of rodent defects for maxillofacial surgery can be found. However, their relevance for further application is questionable. The most well-established defect models performed in rat maxillae are mid-palate cleft and alveolar cleft defects (Nguyen et al., 2009; Toyota et al., 2021). These models are limited to cleft palate research (Mostafa et al., 2014). Another well-described model in rat maxillary is the tooth extraction model that relies on the evaluation of alveolar bone reconstruction after tooth extraction. This model also shows some limitations. First, the size of the rat alveolar bone defect varied a lot between studies without consensus, and the volume of bone to regenerate may vary between animals (Kandalam et al., 2021; Shi et al., 2022; He et al., 2023; Gu et al., 2022; Ni et al., 2022). Depending on the site of extraction [incisor (Shi et al., 2022; He et al., 2023) or first molar (Gu et al., 2022; Ni et al., 2022)], investigation of alveolar bone reconstruction may be challenging to analyze. Alveolar bone induced problems in its quantification because of the difficulty in distinguishing it from the surrounding dental and bone tissues (Park et al., 2007). In addition, only late timepoint (i.e. 12 weeks) was investigated for histological analysis to evaluate new bone synthesis. The effect of the periodontal ligament on alveolar bone regeneration could not be assessed due to a lack of information at intermediate histological timepoints. However, periodontal ligament is known to play a role on bone tissue formation.

To restore the initial curvature of the maxillae, the design of specific biomaterials should consider both the repair volume and the shape of the bone (Liu et al., 2019). The second aim of our study was to test relevant materials that mimic clinical conditions of GBR procedures by combining a bone graft substitute with a membrane (Bottino et al., 2012; Aprile et al., 2020). We used injectable microbeads made from pullulan and dextran and combined with hydroxyapatite (GLYCOBONE) as a bone filling material. GLYCOBONE was selected to fit the defect area, and it induced bone regeneration. These microbeads were already tested in small [rat (Schlaubitz et al., 2014; Maurel et al., 2021; Ribot et al., 2017)] and large [sheep (Fricain et al., 2018; Maurel et al., 2021)] mammal models. Great osteoconductive properties were evidenced with newly formed bone around the microbeads. To improve their efficacy, a membrane with a similar formulation was developed (Ahmed et al., 2023), and then applied here to this bone defect.

Two membranes intended for GBR procedures were thus tested in this model: an already commercialized (BIO-GIDE), and another one under development (GlycoMembrane) (Ahmed et al., 2023). These membranes need to keep their whole integrity at least 16–24 weeks to maintain their barrier function required by GBR procedures for bone ingrowth (Solomon et al., 2022). Micro-CT acquisitions enabled in this study to follow the progressive mineralization of GLYCOBONE (bone filling material) as well as GlycoMembrane (membrane that covered the defect), as they demonstrated similar composition and were radiolucent at T0. These two materials could clearly be distinguished on the 2D scan coronal sections *in vivo* enabling to register the bone formation inside the defect. However, this follow-up could not be performed for BIO-GIDE since no mineralization of this membrane was expected. Quantitatively, BVF ratio for BTE material implantation doubled at the endpoint compared to the defect left empty (BVF around 80% for defect filled with bone substitute vs. BVF under 40% for defect left empty). Qualitatively on histological sections, disorganized bone structures with woven bone was evidenced on defects left empty compare to defects filled with GLYCOBONE. Previous studies already highlighted the osteoconductive role of GLYCOBONE that drive collagen fibers orientation around the microbeads (Figure 7) (Fricain et al., 2018; Schlaubitz et al., 2014). Micro-CT analysis showed similar results whatever the membrane used to cover the bone substitute, except at 6 weeks where BVF was significantly increased with the polysaccharide membrane compared to the collagen one. BIO-GIDE, as well as GlycoMembrane, demonstrated good tissue integration in the defect area and retained the bone filling materials inside the defect, as demonstrated by histological analysis. As longitudinal radiological follow-up was performed, histological analysis could only be achieved at the final timepoint (*i.e.,* 12 weeks), thereby preventing to quantify membranes resorption over time by histomorphometric analysis. However, few important data could be highlighted. First, a large cellularization of the BIO-GIDE membrane was observed which suggesting a faster resorption of the material before the required timepoint for GBR procedures (*i.e.*, 16–24 weeks accordingly to bone healing time). In parallel, a thin fibrotic layer appeared around GlycoMembrane, but the membrane kept its integrity until the last timepoint. Our previous study already demonstrated the biocompatibility of GlycoMembrane with no inflammatory cells around the membrane that remained stable for up to 16 weeks in a subcutaneous model implantation in rats (Ahmed et al., 2023). This study showed here that this new polysaccharide membrane (GlycoMembrane) was as effective as the commercial and widely used collagen membrane to promote bone formation. GlycoMembrane played a crucial role in supporting bone ingrowth and acting as a physical barrier to prevent soft tissue infiltration and retain the bone substitute beads inside the defect area.

The preclinical model implemented here highlighted great interest to mimic GBR procedure in order to achieve first-stage screening, by excluding candidate materials with unfavorable performances. The possibility to combine a bone graft substitute and a GBR membrane was evidenced in this study to investigate their efficiency in alveolar bone regeneration. The present model provides a suitable environment to test new medical devices, and at the same time considers the mechanical forces and load-bearing capacity applied to such devices. Indeed, this model closely mimics the clinical situation in maxillofacial surgery, where bone graft substitutes are mainly used in tooth-bearing areas subject to functional forces from chewing and jaw movements.

Two main limitations of this study must be mentioned concerning radiographic and histological analyses. Firstly, challenges concerning longitudinal follow-up using micro-CT analysis up to 12 weeks were encountered. Proper handling of animals was needed in order to obtain repeatable acquisitions and good image quality (Chatterjee et al., 2017; Ford et al., 2003; Boone et al., 2004). Additionally, it was difficult to differentiate incisor root from bone in the scans due to their similar gray values. The use of the “growing seeded tool brush” during the analysis was thus required to accurately delineate the incisor root and exclude its signal contribution to the BVF. Secondly, histomorphometric analysis of new bone formation was not pursued due to the complexity to underline the edges of the defects, especially in the region between the tooth root and the inferior part of the alveolar bone. This could be explained by the late histological analysis. Indeed, this investigation was limited to a single timepoint for histological analysis (final endpoint - 12 weeks) to minimize animal use, where the mature bone was already synthesized, making it difficult to visualize the limits between native and newly formed bone. Therefore, intermediate timepoints (*e.g.*, 4 and 8 weeks) could be considered for further histomorphometric quantifications (Kumar et al., 2022).

## 5 Conclusion

This study established a standardized bone defect model in rat maxillary for assessing biomaterials in oral and maxillofacial surgery. The 3.3 mm and 4.5 mm defects did not enable to obtain reproducible model to investigate alveolar bone regeneration. The 2.8 mm diameter defect provided reproducibility, and the position of the defect in the maxilla enabled the investigation of alveolar bone regeneration while considering mechanical forces and load-bearing capacity. The use of this rat model for maxillary bone defect allows an ethically aligned approach and minimizes invasiveness compared to existing models. Furthermore, this new bone defect model was validated to test innovative biomaterials for GBR applications in oral and maxillofacial surgery, as demonstrated by the successful implantation and analyses of a bone substitute covered by two different membranes. The development of this standardized small animal model should thus be considered as a first step in the evaluation and validation of new biomaterials for bone regeneration in oral and maxillofacial surgery before the use of larger mammals or clinical study.

## 6 Statement of significance

The investigation into innovative biomaterials for guided bone regeneration (GBR) in oral and maxillofacial surgery is imperative for advancing clinical outcomes. This study addresses a critical gap by proposing and validating a reproducible rat maxillary bone defect model, offering a valuable platform to evaluate novel materials for GBR

## Data Availability

The raw data supporting the conclusions of this article will be made available by the authors, without undue reservation.
